# Massachusetts’ innovative policy approach to expanding contraceptive options in primary care

**DOI:** 10.1093/haschl/qxad035

**Published:** 2023-08-24

**Authors:** Chloe Ciccariello, Viveka R Prakash-Zawisza, Lydia E Pace

**Affiliations:** Boston Medical Center, Section of General Internal Medicine, Boston, MA 02118, United States; Boston University, Boston, MA 02118, United States; Massachusetts Executive Office of Health and Human Services, MassHealth, Boston, MA 02108, United States; University of Massachusetts Chan Medical School, Department of Obstetrics and Gynecology, Worcester, MA 01605, United States; Connors Center for Women's Health and Gender Biology, Brigham and Women's Hospital, Boston, MA 02115, United States; Harvard Medical School, Boston, MA 02115, United States

**Keywords:** health policy, value-based care, reproductive justice

## Abstract

As health systems pivot toward value-based care and as the reversal of Roe vs Wade has significantly decreased access to abortion care in the United States, contraception is increasingly recognized as a high-value health service. However, the United States has a long and troubling history of using contraceptive policies and practices, including forced sterilization, to limit the reproductive rights of people of color and individuals with disabilities. We hope to highlight an innovative program developed by Massachusetts’ Medicaid program, which seeks to expand access to long-acting reversible contraception (LARC) within primary care clinics in a way that promotes both value and reproductive justice. This program provides financial incentives for clinics that serve patients with Medicaid to offer LARC to all patients within the primary care space. Unlike LARC programs that exclusively target patients with Medicaid insurance and provide incentivizes based on number of LARC insertions, this policy has the potential to “lift all boats” and expand access to LARC for all patients regardless of payer. Careful evaluation of this program will be necessary to ensure that the intended outcomes—to increase access to LARC, promote reproductive justice, and deliver value to the health system—are achieved.

Contraception is an essential health care service, and access to comprehensive contraceptive care is fundamental to population health and the well-being and autonomy of people who can become pregnant. The erosion of abortion rights in the United States also places a spotlight on the importance of reliable contraception. As health systems pivot toward a focus on value-based care, facilitating access to the full range of contraceptive methods in primary care settings, including long-acting reversible contraception (LARC; ie, intrauterine devices [IUDs] and implants) should be a priority. However, the United States has a long and troubling history of using contraceptive policies and practices, including forced sterilization, to limit the reproductive rights of people of color and people with disabilities.^1^ Policies that promote contraceptive access and uptake must be thoughtfully implemented and critically evaluated to ensure that they advance patient autonomy and reproductive justice.^2^ Massachusetts’ Medicaid program (MassHealth) is now incentivizing LARC availability within an innovative primary care value-based contracting mechanism. If successfully implemented, this novel approach could expand access to LARC within primary care in a way that promotes both value and reproductive justice.

LARC methods are the most effective contraceptive methods available^3^ and increasingly selected by clinicians and patients. Although LARC use in the United States has increased,^4^ many experts believe that LARC remains underutilized.^5^ Successful LARC programs do not stand alone, but rather are one part of a larger sexual and reproductive health landscape that centers the individual patient and their goals.^6^ Offering LARC within primary care can help realize this vision, but a persistent barrier is the insufficient numbers of primary care clinicians and facilities offering these methods.^7^ Limited availability in primary care reflects a lack of training and credentialing standards,^8^ limited institutional support, up-front expense of LARC devices coupled with relatively low reimbursement, and the challenge of incorporating an additional service into overburdened primary care practices.^9^

For decades, various national- and state-level policies have sought to reduce barriers to contraceptive care; several recent initiatives have focused on LARC specifically. Although some policies, including the Affordable Care Act, have mandated coverage of contraception by commercial plans, most initiatives have focused on contraceptive coverage for low-income individuals. Since 1972, the federal government has required Medicaid to cover contraception^10^ and matches funding for these services at a higher rate than other services. Many states additionally use a 1115 waiver, a tool that states can use to innovate on the way they deliver care within their Medicaid programs, to expand coverage for family planning services to individuals who may not be otherwise eligible for these benefits. Title X is a federal grant program that specifically funds family planning and other preventive services for the un- and underinsured.^11^ More recent state-level initiatives have sought to address practice- and provider-level barriers to LARC. To incentivize immediate postpartum LARC, many state Medicaid agencies have now unbundled reimbursement for LARC from global delivery fees.^12^ In a broader initiative, the Delaware Contraceptive Access Now (DelCAN) program, the state of Delaware partnered with the organization UpStream to increase LARC access, primarily targeting Medicaid-insured individuals.^13^ This initiative unbundled immediate postpartum LARC reimbursement from Medicaid global delivery fees, increased funding for LARC devices, and provided clinician and staff training at federally qualified health centers and Title X–funded clinics, and launched public awareness campaigns.

Programs focused on contraceptive and LARC access for low-income individuals, including through Medicaid, are critical to facilitate reproductive choices among underserved communities. Medicaid serves individuals experiencing poverty who also face higher rates of unintended pregnancy and adverse pregnancy outcomes and who also are disproportionately denied access to abortion care around the United States. However, lower-income individuals are also at higher risk of coercive contraceptive practices, including pressure to get and/or keep LARC,^14^ and lower quality contraceptive care. Medicaid family planning policies and other initiatives focused on low-income individuals have rightfully faced criticism, including for a narrow-minded vision of LARC as a “poverty cure.”^15^ LARC methods are not the best fit for everyone, and LARC specifically can be wielded as a tool for reproductive coercion since removal must occur at an appointment with a trained clinician. Policies that center LARC as the best option for certain populations can fuel providers’ pre-existing biases,^16^ precluding patient-centered counseling, eroding trust, and potentially leading to reproductive coercion. To promote reproductive justice, policies need to center patients’ own goals and values in their approaches to family planning.

MassHealth recently received approval for a new 1115 waiver that makes significant changes to the way primary care clinics are reimbursed to incentivize a set of patient-centered services, including LARC.^17^ Under a new primary care sub-capitation program, MassHealth will pay primary care practices a tiered per-member-per-month (PMPM) rate atop a base rate based on the availability of certain services and providers at that practice. These include services that have been under-incentivized in a traditional fee-for-service payment model but may improve health outcomes and decrease costs in the long run, including integrated behavioral health, substance use services, and on-site availability of LARC. The additional reimbursements range from $5 PMPM up to $13 PMPM per practice. To get the highest PMPM rate, clinics must offer both IUDs and implants on-site at least twice a month.

MassHealth's inclusion of LARC in the primary care sub-capitation program is innovative in several ways. First, the inclusion of LARC establishes LARC as a key primary care service alongside other services that are increasingly recognized as fundamental primary care services. Many primary care sites, particularly those staffed by internists (as opposed to family practice clinicians, who more frequently perform procedures), do not offer LARC. LARC is not included as a key competency in internal medicine primary care residency programs, despite growing recommendations for LARC training in internal medicine.^18^ Incentivizing, and expecting, LARC availability in primary care could help catalyze training initiatives and standardize credentialing procedures and standards.

Second, unlike the DelCAN initiative, the MassHealth program does not provide technical support or prescribe how services should be implemented. This will allow clinics to adopt highly individualized approaches to LARC integration that adapt to their own structure, schedules, and patient populations. For example, 1 clinical site may choose to use their extra PMPM payments to train existing staff in LARC placement. Another may choose to “float” a clinician from another site to their clinic to meet this requirement. Although this individualized approach allows for further innovation at the level of the individual practice, it also places the logistical burden of adding this service on already-strapped primary care leadership and staff. This burden is heaviest on smaller, leaner, independent practices and lighter on academic practices or those within multispecialty networks.

We believe that incentivizing the availability of LARC services represents a more equitable and reproductive justice–oriented approach than policies that increase reimbursement for device insertions or impact only Medicaid-insured individuals. By incentivizing the availability of LARC, rather than LARC insertions themselves, the sub-capitation program promotes expanded options for all patients without incentivizing coercive contraceptive counseling. Further, once a clinic has this service available, it can be utilized by patients with all types of insurance, highlighting the powerful phenomenon that policies aimed at elevating the most vulnerable people in society ultimately are of benefit to everyone.

The effectiveness of this policy in enhancing LARC access and reproductive choice will depend on how it is implemented by primary care practices, and this will be important to study. First, it will be important to see whether increased reimbursement will sufficiently incentivize practices to overcome the challenge of LARC training and new workflows. Second, the implementation strategies used, and their success, should be examined to identify the most effective processes for a given setting. Third, the quality and quantity of contraceptive care provided, equity in availability and service delivery within and between practices, availability and provision of other methods, the timeliness of LARC insertion or removal after a patient's request, the quality and patient-centeredness of counseling, and patient satisfaction must be examined. The quality of contraceptive counseling, in particular, is challenging to measure, but reproductive justice–centered strategies to do so have been developed.^19^ We envision a multiyear study that involves both quantitative measurements (eg, claims data for LARC services, number of credentialed LARC providers within primary care settings) and qualitative assessments such as patient and provider surveys, structured interviews, and focus groups. Since the waiver could impact care for patients with any type of insurance, evaluation should focus on LARC services across all payers.

Contraception is only 1 component of reproductive justice–oriented health care: payers and practices must also prioritize programs addressing social determinants of health, high-quality maternity and postpartum care, and fertility services. However, innovative strategies to advance patient-centered comprehensive contraceptive care in primary care are much needed, particularly in the era of value-based care. If well implemented, MassHealth's prioritization of this content area within its new waiver could serve as a model for Medicaid programs across the country.

## Supplementary Material

qxad035_Supplementary_Data
